# Dyarrheal Syndrome in a Patient Co-Infected with *Leishmania infantum* and *Schistosoma mansoni*

**DOI:** 10.1155/2012/240512

**Published:** 2012-11-18

**Authors:** Gláucia Fernandes Cota, Luciana Inácia Gomes, Bruna Fernandes Pinto, Joanna R. Santos-Oliveira, Alda Maria Da-Cruz, Moisés Salgado Pedrosa, Wagner Luiz Tafuri, Ana Rabello

**Affiliations:** ^1^Hospital Eduardo de Menezes, Fundação Hospitalar do Estado de Minas Gerais (Fhemig), Rua Dr Cristiano Resende, 2213, 30622-020 Belo Horizonte, MG, Brazil; ^2^Laboratório de Pesquisas Clínicas, Centro de Pesquisas René Rachou, Instituto Oswaldo Cruz-FIOCRUZ, Avenida Augusto de Lima, 1715, 30190-002 Belo Horizonte, MG, Brazil; ^3^Laboratório Interdisciplinar de Pesquisas Médicas, Instituto Oswaldo Cruz-FIOCRUZ, Avenida Brasil, 4365, 21040-360 Manguinhos, Rio de Janeiro, RJ, Brazil; ^4^Departamento de Patologia Geral, Instituto de Ciências Biológicas, Universidade Federal de Minas Gerais, Avenida Antônio Carlos, 6627, 31270-901 Belo Horizonte, MG, Brazil

## Abstract

This case report describes an atypical clinical presentation of visceral leishmaniasis affecting the digestive tract and causing malabsorption syndrome in a patient without recognized immunosuppressive condition. After appropriate treatment for the classical visceral form of the disease, diarrhea persisted as the main symptom and massive infection by *Leishmania* was detected by histopathology analysis of the duodenal mucosa. *Schistosoma mansoni* coinfection was also confirmed and treated without impact on diarrhea. New course of amphotericin B finally led to complete improvement of diarrhea. Atypical visceral leishmaniasis involving the gastrointestinal tract is well recognized in HIV coinfection but very rare in immunocompetent patients. The factors determining the control or evolution of the *Leishmania* infection have not been completely identified. This case stresses the importance of atypical symptoms and the unusual location of visceral leishmaniasis, not only in immunodepressed patients, and raises the possible influence of chronic infection by *S. mansoni* reducing the immune response to *Leishmania*.

## 1. Introduction

Visceral leishmaniasis (VL) due to (*Leishmania*) *infantum* (sin *L*. *chagasi*) is endemic in Brazil. The incidence in adult patients has increased in recent years [1]. Typical clinical features are fever, hepatosplenomegaly, hypergammaglobulinaemia, and pancytopenia. Cases of VL have also presented atypically involving the lungs, pleura, oral mucosa, larynx, esophagus, stomach, small intestine, and skin [2, 3]. These atypical cases were described mostly in patients infected with HIV [4, 5] or with any recognized immunosuppressive condition such as diabetes, lymphoma and elderly [3, 6, 7]. It suggested that the clinical manifestations may be influenced by the immunological status of the host.

## 2. Case Report

**Figure 1:**
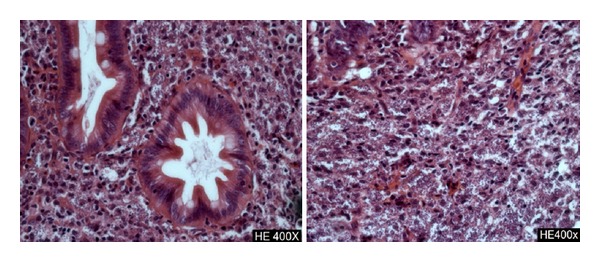
Histological analysis of the duodenum showing extensive infiltration with *Leishmania* bodies (hematoxylin and eosin, ×400).

**Figure 2:**
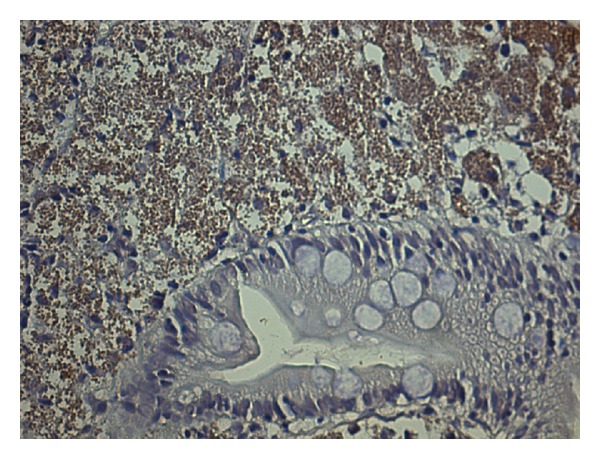
The immunohistochemical study of duodenum fragment tissue confirming *Leishmania* presence.

A 42-year-old man presented with a six-month history of nonbloody diarrhea associated with the clinical triad of fever, splenomegaly, and pancytopenia. Anti-*Leishmania* antibodies serological tests (indirect immunofluorescence title 1 : 640 and rapid test with the recombinant k39) were positive. The diagnosis of visceral leishmaniasis was firmed by the detection of amastigotes in a bone marrow aspirate smears. Because of upper gastrointestinal symptoms (mild dysphagia and vomiting), an esophagogastroduodenoscopy was performed, revealing in duodenum the presence of erosions coated with fibrin, plates of enanthema, and thickened pleats with small whitish spots distributed diffusely. In a tissue fragment obtained by biopsy, the duodenal mucosa showed villous enlarged and filled with numerous histiocytes packed with intracytoplasmic round structures with morphology suggestive of amastigotes of *Leishmania* spp. The patient received liposomal amphotericin B, 4 mg/kg of body weight during 5 days with complete improvement of visceromegaly, fever and recovery of white blood cell and platelets counts. After treatment and hospital discharge, diarrhea and anemia persisted, and his condition continued to deteriorate. Four months later the patient was admitted once again to hospital with worsening diarrhea, dehydration, metabolic acidosis, and electrolyte imbalance. Blood tests revealed a normochromic normocytic anemia, profound hipoalbuminemia (1.9 g/dL), and a high erythrocyte sedimentation rate (120 mm/1 hour). Laboratory examinations also revealed low serum folic acid and iron levels. Both vitamins were replaced and nutritional support was beginning. This time, an extensive search for parasites in stool microscopy was done and eggs of *S*. *mansoni* were identified on stool microscopy. The patient was treated with praziquantel 60 mg/kg of body weight. After four weeks, diarrhea remained unchanged, and his condition continued to deteriorate with anorexia and weight loss. Abdominal computer tomography showed mild splenomegaly, no lymphadenopathy, and a normal liver size. There was mild dilatation of intestinal loops of the colon. A bone marrow aspiration was also performed showing only reactive changes and no *Leishmania* amastigotes. Real-time quantitative PCR (qPCR) assessment on whole blood and bone marrow samples showed positive results using the ribosomal RNA small subunit gene (SSU rRNA) from *Leishmania* spp. as DNA target. Patient's lymphocyte counts were TCD4^+^: 539/mm^3^, TCD8^+^: 503/mm^3^ e TCD3^+^: 1,094/mm^3^. A colonoscopy was normal. Despite the absence of symptoms of upper gastrointestinal system, an upper gastrointestinal endoscopy was performed, and it showed the presence of diffuse pallor in stomach and duodenum with swollen appearance without ulceration. Duodenal mucosa histopathology showed the persistence of amastigotes, as four months before (Figure 1). The immunohistochemical study [8] confirmed the presence of numerous immunolabeled amastigotes forms of *Leishmania* (Figure 2). Tests to detect anti-*Trypanosoma cruzi* antibodies, a protozoan also endemic in Brazil and with tropism for the gastrointestinal tract, and the anti-HIV testing were negative. The patient was treated with amphotericin B deoxycholate, 0.8 mg/kg of body weight for 30 days, and diarrhea resolved completely within two weeks. Two months after the end of treatment the patient had reached its normal weight and normalized erythrocyte levels. The plasmatic levels of IFN-*γ* and IL-10 were measured before and after treatment by sandwich-enzyme-linked immuonosorbent assay, as previously described [9]. The results were expressed as picograms per milliliter (pg/mL) based on a standard curve. IFN-*γ* was undetectable before and after treatment, while IL-10 levels decreased from 36 pg/mL at initial evaluation to 16.3 pg/mL (55% reduction) after amphotericin treatment and clinical improvement.

## 3. Discussion and Literature Review

Gastrointestinal involvement in visceral leishmaniasis has been reported mainly in HIV/AIDS patients. Furthermore, although patients with VL may have symptoms of mild diarrhea, the presence of severe enteropathy, not accompanied by other classic signs of systemic involvement as the presenting feature, is rare: a literature review revealed only four case reports occurring in nonimmunosuppressed patients [7, 10, 11]. There are at least another five confirmed cases of intestinal involvement following the classical form of visceral leishmaniasis in immunocompetent individuals [12–14].

The exact pathogenesis of the diarrhea is not clear, it is suggested that symptoms in enteropathic VL may be a combination of parasitism of the reticuloendothelial cells of the submucosa, bacterial overgrowth, partial villous atrophy, competition between host and parasite for nutrients, altered motility, bile salt deconjugation, and lymphatic blockade [15, 16]. In the present case, as diarrhea does not respond to treatment and elimination of *S*. *mansoni*, it is reasonable to suppose that enteropathy was due to mucosal involvement by *Leishmania* rather than *S*. *mansoni*. On the other hand, the noncomplete response after a first-line treatment as liposomal amphotericin is intriguing. It should be noted that after the eradication of *S*. *mansoni*, another attempt of treatment with amphotericin has achieved an excellent response. In recent years, studies have demonstrated that helminthic infection (including *S*. *mansoni*) or products from their infections are able to downmodulate the type 1 TCD4^+^ T-cell inflammatory response [17, 18]. This temporal association and evidence already available on the immune modulation induced by helminthes encourage us to hypothesize that the interaction between the two agents (*S*. *mansoni* and *Leishmania*) contributed to this unusual manifestation occurrence and to a temporary resistance to treatment.

The factors determining the development of a clinically manifested form of VL (acute, classic, oligosymptomatic or atypical form) have not yet been completely identified. After inoculation of the promastigote form into the skin by the insect vector, a complex interaction between the parasite and the host immune response occurs, and its results can modulate the clinical presentation of VL [19]. With *Leishmania* spp. being recognized as an obligate intracellular parasite of macrophages, studies have demonstrated that the specific immunity in VL is mediated by TCD4^+^ T helper (Th) cells and that disease susceptibility is associated with the inability to produce a macrophage-stimulating cytokine profile (Th1 profile) including interferon gamma (IFN-*γ*) and interleukins (IL)-2 and IL-12, while, on the other hand, an elevated production of immunosuppressive cytokines such as IL-10 and IL-4 (Th2 profile) as well as high levels of tumor necrosis factor-alpha (TNF) is observed [20]. In the reported case, we documented presence of IL-10 in the active phase of the disease but surprisingly, our data showed suppressed levels of IFN-*γ*, which could be due to *S*. *mansoni* infection and explain the massive and deep infection by *Leishmania*. This finding is in agreement with others who demonstrated that the addition of the *S*. *mansoni *antigens to peripheral blood mononuclear cells cultures of patients infected with *L*. *braziliensis *(stimulated with soluble* Leishmania* antigen) reduced IFN-*γ* and TNF production; conversely, these cells produced increased levels of IL-10 [18]. Another pathophysiological mechanism was suggested by an experimental study [21] that showed that *S*. *mansoni*-infected mice fail to control a superimposed *L*. *donovani* infection. The failure occurs despite the development of a functional anti-*L*. *donovani* Th1 response that can mediate granuloma formation and effective clearance of amastigotes from foci of infection in the hepatic parenchyma. Instead, anti-*Leishmania* immunity fails within the *S*. *mansoni* egg granuloma, consistent with a lack of *L*. *donovani* granuloma assembly in this tissue microenvironment and consequent lack of nitric oxide production.

Helminthes employ a range of immunomodulatory strategies to modulate the host immune response and utilize it to extend their longevity in the host and facilitate transmission. Studies of patients with concurrent infections of *L*. *braziliensis* and a helminthes infection have shown that these patients tend to present with smaller ulcers than patients without helminthes infections. However, the time for the lesion to heal was approximately double in coinfected patients [22]. In experimental studies, it has been shown that *S*. *mansoni *infection is able to downmodulate the Th1 inflammatory response that is implicated in several autoimmune diseases, such as type-I diabetes, encephalomyelitis, and psoriasis [23–25]. Moreover, there is evidence that *Schistosoma* sp. infection or its products have the potential to modulate Th2-immune responses that result in the pathology of allergic diseases [26]. Perhaps to control inflammation associated with the passage of *S*. *mansoni* eggs from the vascular compartment to the liver parenchyma, eggs inhibit toll-like receptors (TLRs-) mediated dendritic cells (DCs) activation and activate innate and adaptive immune responses that result in the production of the anti-inflammatory cytokines, IL-4 and IL-10. The importance of these pathways is attested to not only by their crucial role in the survival of schistosome-infected hosts but also in the ability of schistosomes and other helminthes to ameliorate Th1-response-mediated autoimmune pathologies [17]. As an example, asthmatic patients who are infected with *S*. *mansoni* have a less severe course of asthma, and it seems to be mediated by IL-10 [27].

## 4. Conclusion

We describe an unusual presentation of visceral leishmaniasis accompanied by intestinal involvement in an immunocompetent patient. Even with extensive investigation we did not find any immunosuppressive condition, except the *S*. *mansoni* coinfection. This case report might represent an example of the immune modulation induced by *S*. *mansoni* and can add to other evidence in the search for elucidating the complex mechanisms involved in the states of susceptibility and resistance to *Leishmania* infection. In addition, this report also shows that intestinal infection by *Leishmania* spp. must be included in the differential diagnosis for chronic diarrhea among patients with a previous history of leishmaniasis or living in an endemic area.
